# Supplementary material for: Use of Systemic Antibiotics in Patients with COVID-19 in Colombia: A Cross-Sectional Study
Source: Antibiotics (Basel). 2023 Jan 26;12(2):252. doi: 10.3390/antibiotics12020252 (PMC9952122; doi:10.3390/antibiotics12020252)
Supplement: Supplementary file 1 [file antibiotics-12-00252-s001.zip › antibiotics-2130321-supplementary.pdf]

Supplementary Table S1. Classification of antibiotics according to pharmacological group and according to AWaRe.

**Classification by pharmacological group**	
**Aminoglycosides**	Amikacin, Gentamicin
**Carbapenems**	Ertapenem, Imipenen/cilastatin, Meropenem
**Cephalosporins**	Cefadrine, Cephalexin, Cephalotin, Cefazolin, Cefepime, Ceftaroline, Ceftazidime/avibactam, Ceftazidime, Ceftolozano/tazobactam, Ceftriaxone, Cefuroxime
**Fluoroquinolones**	Ciprofloxacin, Levofloxacin, Moxifloxacin
**Glycopeptides**	Vancomycin
**Glycylcyclines**	Tigecycline
**Lincosamides**	Clindamycin
**Lipopeptides**	Daptomycin
**Macrolides**	Azithromycin, Clarithromycin, Erythromycin
**Monobactams**	Aztreonam
**Oxazolidinones**	Linezolid
**Penicillins**	Amoxicillin, Amoxicillin/clavulanic acid, Ampicillin, Ampicillin/sulbactam, Oxacillin, Penicillin G, Piperacillin/tazobactam
**Polymyxins**	Colistin, Polymyxin B
**Sulfonamides**	Trimethoprim/sulfamethoxazole
**Tetracyclines**	Doxycycline, Tetracycline
**Classification AWaRe**	
**Access**	Amikacin, Amoxicillin, Amoxicillin/clavulanic-acid, Ampicillin, Ampicillin/sulbactam, Azidocillin, Bacampicillin, Benzathine-benzylpenicillin, Benzylpenicillin, Brodimoprim, Cefacetrile, Cefadroxil, Cefalexin, Cefaloridine, Cefalotin, Cefapirin, Cefatrizine, Cefazedone, Cefazolin, Cefradine, Cefroxadine, Ceftezole, Chloramphenicol, Clindamycin, Clometocillin, Cloxacillin, Dicloxacillin, Doxycycline, Epicillin, Flucloxacillin, Furazidin, Gentamicin, Hetacillin, Mecillinam, Metampicillin, Meticillin, Metronidazole_IV, Metronidazole_oral, Nafcillin, Nifurtoinol, Nitrofurantoin, Ornidazole_IV, Ornidazole_oral, Oxacillin, Penamecillin, Phenoxymethylpenicillin, Pivampicillin, Pivmecillinam, Procaine-benzylpenicillin, Propicillin, Secnidazole, Spectinomycin, Sulbactam, Sulfadiazine, Sulfadiazine/tetroxoprim, Sulfadiazine/trimethoprim, Sulfadimethoxine, Sulfadimidine, Sulfadimidine/trimethoprim, Sulfafurazole, Sulfaisodimidine, Sulfalene, Sulfamazone, Sulfamerazine, Sulfamerazine/trimethoprim, Sulfamethizole, Sulfamethoxazole, Sulfamethoxazole/trimethoprim, Sulfamethoxypyridazine, Sulfametomidine, Sulfametoxydiazine, Sulfametrole/trimethoprim, Sulfamoxole, Sulfamoxole/trimethoprim, Sulfanilamide, Sulfaperin, Sulfaphenazole, Sulfapyridine, Sulfathiazole, Sulfathiourea, Sultamicillin, Talampicillin, Tetracycline, Thiamphenicol, Tinidazole_IV, Tinidazole_oral, Trimethoprim

**Watch**	Arbekacin, Aspoxicillin, Azithromycin, Azlocillin, Bekanamycin, Biapenem, Carbenicillin, Carindacillin, Cefaclor, Cefamandole, Cefbuperazone, Cefcapene-pivoxil, Cefdinir, Cefditoren-pivoxil, Cefepime, Cefetamet-pivoxil, Cefixime, Cefmenoxime, Cefmetazole, Cefminox, Cefodizime, Cefonicid, Cefoperazone, Ceforanide, Cefoselis, Cefotaxime, Cefotetan, Cefotiam, Cefoxitin, Cefozopran, Cefpiramide, Cefpirome, Cefpodoxime-proxetil, Cefprozil, Cefsulodin, Ceftazidime, Cefteram-pivoxil, Ceftibuten, Ceftizoxime, Ceftriaxone, Cefuroxime, Chlortetracycline, Cinoxacin, Ciprofloxacin, Clarithromycin, Clofoctol, Clomocycline, Delafloxacin, Demeclocycline, Dibekacin, Dirithromycin, Doripenem, Enoxacin, Ertapenem, Erythromycin, Fidaxomicin, Fleroxacin, Flomoxef, Flumequine, Flurithromycin, Fosfomycin_oral, Fusidic-acid, Garenoxacin, Gatifloxacin, Gemifloxacin, Grepafloxacin, Imipenem/cilastatin, Isepamicin, Josamycin, Kanamycin_IV, Kanamycin_oral, Lascufloxacin, Latamoxef, Levofloxacin, Levonadifloxacin, Lincomycin, Lomefloxacin, Loracarbef, Lymecycline, Meropenem, Metacycline, Mezlocillin, Micronomicin, Midecamycin, Minocycline_oral, Miocamycin, Moxifloxacin, Nemonoxacin, Neomycin_IV, Neomycin_oral, Netilmicin, Norfloxacin, Ofloxacin, Oleandomycin, Oxolinic-acid, Oxytetracycline, Panipenem, Pazufloxacin, Pefloxacin, Penimepicycline, Pheneticillin, Pipemidic-acid, Piperacillin, Piperacillin/tazobactam, Piromidic-acid, Pristinamycin, Prulifloxacin, Ribostamycin, Rifabutin, Rifampicin, Rifamycin_IV, Rifamycin_oral, Rifaximin, Rokitamycin, Rolitetracycline, Rosoxacin, Roxithromycin, Rufloxacin, Sarecycline, Sisomicin, Sitafloxacin, Solithromycin, Sparfloxacin, Spiramycin, Streptoduocin, Streptomycin_IV, Streptomycin_oral, Sulbenicillin, Tazobactam, Tebipenem, Teicoplanin, Telithromycin, Temafloxacin, Temocillin, Ticarcillin, Tobramycin, Tosufloxacin, Troleandomycin, Trovafloxacin, Vancomycin_IV, Vancomycin_oral
**Reserve**	Aztreonam, Carumonam, Cefiderocol, Ceftaroline-fosamil, Ceftazidime/avibactam, Ceftobiprole-medocaril, Ceftolozane/tazobactam, Colistin_IV, Colistin_oral, Dalbavancin, Dalfopristin/quinupristin, Daptomycin, Eravacycline, Faropenem, Fosfomycin_IV, Iclaprim, Imipenem/cilastatin/relebactam, Lefamulin, Linezolid, Meropenem/vaborbactam, Minocycline_IV, Omadacycline, Oritavancin, Plazomicin, Polymyxin-B_IV, Polymyxin-B_oral, Tedizolid, Telavancin, Tigecycline

Supplementary Table S2. Binary logistic regression of the variables related to receiving antibiotics Watch/Reserve in 10,916 patients with a diagnosis of COVID-19 treated in 8 highly complex clinics in Colombia.

Variables	Sig.	OR	CI95%	
			Lower	Upper
Men	<0.001	1.437	1.304	1.584
Age ≥65 years	<0.001	1.521	1.358	1.702
Origin Caribbean Region	<0.001	3.078	2.691	3.521
Dyspnea on admission	<0.001	1.315	1.180	1.464
Rheumatoid arthritis	0.473	1.176	0.756	1.828
Ischemic heart disease	0.126	0.804	0.609	1.063
Diabetes mellitus	0.013	1.174	1.035	1.332
Chronic obstructive pulmonary disease	0.047	0.804	0.648	0.997
Chronic kidney disease	0.384	0.897	0.701	1.146
Arterial hypertension	<0.001	1.493	1.335	1.669
First 10 months of the pandemic	<0.001	2.186	1.974	2.422
Emergency Care	<0.001	Reference	Reference	Reference
Hospital Care	<0.001	3.596	3.194	4.048
Intensive Care Unit	<0.001	8.014	6.815	9.423
Systemic corticosteroids	<0.001	3.257	2.903	3.654
Vasopressors - inotropes	<0.001	1.919	1.531	2.405
Invasive mechanical ventilation	<0.001	2.300	1.842	2.870

Sig: Statistical significance; OR: Odds Ratio; CI: Confidence interval
